# Examining the association between the Mediterranean diet and depression: a cross-sectional study in Lebanon

**DOI:** 10.3389/fnut.2025.1692981

**Published:** 2025-12-17

**Authors:** Rana F. Abdel Sater, Rudy S. Younes, Sofi G. Julien

**Affiliations:** 1 Faculty of Arts and Sciences, Department of Nutrition and Food Sciences, Holy Spirit University of Kaslik, Jounieh, Lebanon; 2 Faculty of Arts and Sciences, Department of Psychology and Social Sciences, Holy Spirit University of Kaslik, Jounieh, Lebanon

**Keywords:** Mediterranean diet, depression, social factors, nutritional psychology, mental health, cultures

## Abstract

**Introduction:**

Sociocultural practices, including traditional dietary patterns, influence mental health and depression. The Mediterranean diet (MD), which is common in many Middle Eastern countries such as Lebanon, is regarded as one of the healthiest dietary patterns available. Although the link between MD and mental health is gaining recognition, there is still a lack of substantial evidence to support this relationship.

**Objectives:**

This study sought to examine the relationship between depression and adherence to MD (AMD).

**Methods:**

A cross-sectional study was conducted among 525 participants through an online questionnaire that assessed the severity of depression using the Patient Health Questionnaire 9 (PHQ-9), AMD using the Mediterranean Diet Adherence Screener (MEDAS) questionnaire, and relevant sociodemographic variables. Descriptive, linear regression, and bivariate logistic regression analyses were performed to answer the research questions.

**Results:**

24% of participants self-reported depressive symptoms, as indicated by a PHQ-9 score of 10 or higher, with the majority being female. The scores on the MEDAS and the PHQ-9 were significantly different between the groups classified as having depression and those without depression (7.4 ± 2.4 vs. 7.9 ± 2.5; *p* = 0.033) and (14.2 ± 4 vs. 5 ± 2.6; *p* < 0.001), respectively. The MEDAS score was inversely correlated with the PHQ-9 score (*r* = −0.126, *p* = 0.003), indicating a relationship between depression and AMD. The adjusted model indicated that consuming two or more servings of vegetables per day is associated with a decrease in the likelihood of experiencing depressive symptoms (*b* = −0.66, *p* = 0.021). Several social factors, including employment status, education level, income, and marital status, have been identified as predictors of depression.

**Conclusion:**

Sufficient vegetable consumption may help reduce the risk of depression. These findings could have important implications for mental health prevention and treatment.

## 1 Introduction

There is growing global evidence that poor dietary habits have profound negative influences on the development of non-communicable diseases (NCDs) (1, 2). Among them, depression, a multifactorial mental disorder, is becoming a serious global burden, with more than 340 million affected, according to the latest estimations (3). It is estimated to rank first of the NCD burden by 2030, according to the World Health Organization, and even to surpass physical illnesses (4). Depression is especially salient in Lebanon, as the cumulative crises that this country has experienced, from wars and invasions to a recent severe economic crisis, have led to a substantial rise in mental disorders (5, 6). A recent national prevalence study by Karam et al. (7) found that almost half of Lebanese people suffer from depression. Many hypotheses attempted to explain how depressive disorders develop, including the role of inflammation, genetic and environmental factors, changes in the brain structure and function, social and psychological influences, low levels in monoamine-derived neurotransmitters, dysfunction in the gut-brain axis, poor gut microbiota, and lifestyle choices like diet (8, 9). This growing understanding of the multitude of factors influencing the risk and progress of depression and mental disorders has led to the emergence of fields like social psychiatry and nutritional psychiatry, which explore dietary patterns and social factors and their effect on mental health (10, 11).

The Mediterranean diet (MD) is a traditional healthy dietary pattern specifically adopted by some coastal countries of the Mediterranean Sea (Lebanon, Italy, Greece, Spain, Turkey, Cyprus, and Egypt) that has long featured greater health outcomes compared to the Occidental countries that are relying on a more westernized dietary pattern (12, 13). Beyond its nutritional benefits, the MD represents a crucial part of the Lebanese cultural heritage. It is rooted in traditional practices that have been passed down through generations. This diet is an integral part of social events in Lebanon, traditionally consumed during communal meals that range from family gatherings to rituals designed to reinforce social bonds and community connections (14). The MD is recognized for its richness and accessibility to a variety of fruits, vegetables, and fish, from which olive, avocado, and fish oils can be produced. It also features a low intake of processed food and saturated fat. More specifically, a good adherence to the MD (AMD) is characterized by an adequate intake of unrefined whole grains (barley, bulgur, freekeh, rice, spelt, farro, buckwheat, oat); legumes and pulses (chickpeas, beans and lentils); fatty fish (salmon, anchovies, mackerel, tuna, herring or sardines); the use of extra-virgin olive oil as the main fat source; nuts (pine nuts, pistachios, almonds, walnuts, pecans, cashews) and seeds (sunflower, pumpkins, sesame, chia, flaxseed); a moderate intake of dairy products (cow or goat milk, yogurt like Laban, or labneh, and cheese such as feta, parmesan, halloumi, ricotta, mozzarella); red wine; and a low intake of processed foods and non-stimmed red meat (beef, lamb, pork or veal); and sofrito seasoning with herbs and spices (garlic, onions, peppers, oregano, basil, thyme, rosemary, cumin, coriander, cinnamon) (15, 16). Compliance with MD, especially from a high intake of fruits, vegetables, legumes, nuts, fish, and olive oils, allows consumers to receive numerous health benefits from adequacy in the bioavailability of essential amino acids, essential fatty acids, polyphenols, fiber, minerals, and vitamins (17, 18). These nutrients work synergistically to support overall health and reduce the risk of chronic diseases (9, 19).

Although a good AMD requires a sustained and adequate intake of its food and their specific nutrients, the compliance with MD varies considerably across the countries of the Mediterranean Basin depending on the sociocultural, geographical, and economic influences (20). Therefore, the specific contribution of food groups to depressive symptoms vary among countries due to regional variations in the AMD and are sometimes difficult to identify. In Italy, it was recently demonstrated that the consumption of lipids rich in polyunsaturated fatty acids and low in saturated ones was inversely associated with depressive symptoms (21, 22). Depression in adults was found to be inversely associated with a high vegetable and low alcohol intake in Greece and Portugal (23, 24); and with a high intake of fish and fruit, along with limited consumption of sugar-rich foods or red meat in Spain (25). The MEDAS score was positively associated with the overall well-being in Portugal (26), Greece and Cyprus (27). Similarly, among adolescents and young adults, a high AMD was associated with a reduced likelihood of experiencing depressive symptoms in Spain (28) and Turkey (29). Finally, in a French cohort, an increased consumption of ultra-processed foods has been linked to a higher risk of depression among young adults. Conversely, a diet rich in fruits, nuts, and green vegetables was associated with a lower risk of depression (30).

Despite the growing body of evidence supporting the beneficial effects of satisfactory compliance with the MD or any related plant-based diets on mental health, specifically depressive symptoms, some studies have produced inconclusive results (31). This raises controversy on this trending topic (32, 33) and highlights a lack of information regarding the specific food types that contribute to improved outcomes of depression (26, 27). AMD could explain some of the variations in depression risk, but it is important to consider other factors as potential determinants of depression. Social factors such as socioeconomic status, financial stability, employment status, and educational level are particularly salient in Lebanon, given the cumulative crises the country has faced. These factors may further influence this relationship and contribute to the overall risk for depression in the Lebanese population (6).

Thus, this research aims to evaluate the conditions of depressive symptoms within Lebanon’s social context by examining dietary and other social variables. The objectives of our study are (1) to confirm the negative association between AMD and the risk of depression, (2) to identify specific food components of the MD that may lower the risk for depression among adult Lebanese individuals, and (3) to explore additional social factors that may serve as risk factors for depression.

## 2 Methods

### 2.1 Ethical consideration

The study was conducted in accordance with the Declaration of Helsinki and obtained ethical approval from the Research Ethics Committee of the Higher Center for Research at Holy Spirit University of Kaslik (protocol code HCR/EC 2023/07). The participants provided a digital informed consent to participate in this study.

### 2.2 Sample and study design

The expected prevalence (P) of depression was estimated at 33.4% based on the most recent previous study (34). Precision (*d*) was set at 5%, and the value of *Z* was 1.96 at the 95% level of confidence. The ideal sample size was therefore calculated to be a minimum of 342 participants using the Cochran’s formula.


$$n=\frac{Z^{2}P(1-P)}{d^{2}}$$

A total of 633 persons accessed the survey, and 108 of them were excluded based on the following exclusion criteria: declined participation (*n* = 8); older than 65 (*n* = 6) or younger than 18 (*n* = 8); non-Lebanese citizen (*n* = 23); Lebanese citizen but not resident in Lebanon (*n* = 23); and those suffering from medical conditions requiring medication, such as cardiovascular disease, depression, or anxiety, that could potentially bias the results of the data analysis (*n* = 22). Finally, 525 participants were included in this study based on the inclusion criteria: agreed to participate, age between 19 and 64, Lebanese citizens residing in Lebanon, and not suffering from any medical condition.

### 2.3 Data collection

The survey comprised an English questionnaire designed to gather sociodemographic, dietary and mental health information, created in Google Forms, and disseminated via the snowball sampling method through social media platforms such as WhatsApp, Facebook, Instagram *via* an URL link as previously outlined by our group (35–37). All data were collected from April to July 2023.

#### 2.3.1 Assessment of the adherence to the Mediterranean diet

The AMD was assessed using the validated Mediterranean Diet Adherence Screener (MEDAS) (38). This questionnaire consists of 14 questions, including 12 pertaining to food items and 2 regarding food preferences. Each question answered “Yes” was scored 1 point, while a “No” was not granted any point, resulting in a total score ranging from 0 to 14 for the questionnaire (39). The adherence levels to the MD were classified as low, average, and high, corresponding to scores of 0–5, 6–9, and 10–14, respectively.

#### 2.3.2 Assessment of depression

The severity of depressive symptoms was estimated using the validated short Patient Health Questionnaire (PHQ-9) (40). The scale scores were computed as the sum of the nine items, which can range from 0 to 27. For the analysis, the PHQ-9 score was dichotomized into binary variables with a cut-off point of 10 as previously published (41, 42). Participants with a score of less than 10 were therefore considered not depressed and were classified as the group “None,” while those with a score of 10 or higher were classified as depressed and were included in the group “Depression.” The PHQ-9 has been validated in a Lebanese sample and has shown good psychometric properties (43).

#### 2.3.3 Sociodemographic and other characteristics

The continuous variables age and body mass index (BMI) and the categorical variables (education, income, living status, demographic, region, marital status and smoking status) were collected and used as covariates in this study. All these variables, along with previous one MEDAS and PHQ9 are presented in Supplementary Table S1 with their corresponding measurement scales and coding scheme. The participants were asked to select their body weight in kilograms and height in centimeters from a dropdown list. The self-reported data were then used to estimate the BMI based on these reported anthropometric measures. The formula used to calculate the estimated BMI was the total body weight in kilograms divided by the square of the height in meters. The BMI was classified into 4 categories according to the World Health Organization and as previously published (35) as “underweight,” “normal,” “overweight,” and “obese” with BMI in kg/m^2^ less than 18.5, between 18.5 and 24.9, more than or equal to 25, and more than or equal to 30, respectively.

### 2.4 Statistical analysis

Categorical variables were described by frequencies and percentages, and continuous variables by the median and a standard deviation (SD). A Kolmogorov–Smirnov test was used to assess whether age, BMI, PHQ-9, and MEDAS data followed a normal distribution, in addition to the visualization of the quantile-quantile (Q-Q plots) graphical method. A *p*-value < 0.05 indicated that the data were not normally distributed; therefore, non-parametric tests were used. Comparison of the categorical variables between the two independent groups of depression conditions, “Depression” and “No depression,” was done using the chi-square test for the categorical variables and the Mann–Whitney U-test for the continuous variables. Spearman’s rank correlation was used to measure the direction and the strength of the linear relationship between the MEDAS scoring and the PHQ-9 score. Logistic regression models were presented as odd ratios with a 9% confidence interval, and the degree of changes in depressive symptoms for every one-unit change in the items of MEDAS was expressed with the beta coefficient (Coef. B). *p* values (*p*) less than 0.05 were considered significant for all tests presented in this study. The statistical analyses were performed using GraphPad Prism 10.

## 3 Results

### 3.1 Sociodemographic and anthropometric characteristics of the participants

A total of 525 participants met the eligible criteria and completed the questionnaire and were enrolled for the data analyses. The sociodemographic and other characteristics of our study group are presented in Table 1. The mean age was 27.7 ± 9.7, and most participants were from the Mount Lebanon region (79%), female (58%), employed full-time (44.4%), single (76%), and lived with their parents (74%). Most of them reached a high education level above the undergraduate studies (86.2%), with the minimum salary limit below $500 as being the most prevalent (61%). The BMI was in the healthy category for 57% of the participants, with a mean of 24.5 ± 4.6. Most of them adopted a free EC-smoking attitude (60%). The mean scores for the MEDAS and PHQ-9 were 7.8 ± 2.5 and 7.2 ± 4.9, respectively.

**Table 1:** Sociodemographic and other characteristics of the study sample (*n* = 525).

Variables	Frequency	Percent
Sex		
Female	304	57.9
Male	221	42.1
Region		
Beirut	54	10.3
Beqaa	16	3
Mount Lebanon	414	78.9
North Lebanon	34	6.5
South Lebanon	7	1.3
Marital status		
Single	399	76
Married	117	22.3
Divorced/Separated/Widowed	6	1.7
Living status		
Live alone	35	6.7
Live with another person	102	19.3
Live with parents	388	74
Education level		
Elementary school and below	15	2.8
High school	56	11
College / undergraduate and above	454	86.2
Current occupation		
Employed for full-time job	233	44.4
Employed for part-time job	61	11.6
Retired	6	1.1
Studying	191	36.4
Monthly income		
Less than $500	320	61
$500–$1,000	78	14.9
More than $1,000	127	24.2
Electronic cigarette smoking status		
Smoker	139	26.5
Nonsmoker	315	60
Never heard about electronic cigarette	71	13.5
Body mass index categories		
Underweight	27	5.1
Healthy	299	57
Overweight	138	26.3
Obese	61	11.6
Depressive symptoms		
Depression	127	24.2
None	398	75.8

	Median	SD
Age	24.0	9.7
Body mass index	23.8	4.6
MEDAS score	8	2.5
PHQ9 score	6	4.9

MEDAS (Mediterranean Diet Adherence Screener); PHQ9 (Patient Health Questionnaire 9).

### 3.2 Characteristics of the participants by mental health condition based on a PHQ-9 score >10

Table 2 presents the associations between the sociodemographic characteristics of the participant who self-reported depressive symptoms, categorized as “Depression” or “No depression,” based on a PHQ-9 score greater than or equal to 10 or less than 10, respectively. We observed that marital status, current living status, education, employment, monthly income, and age score were significantly associated with depression. The mean score of the PHQ-9 was higher and statistically different between people presenting depressive symptoms and those without mental health conditions (14.2 ± 4 vs 5 ± 2.6, respectively; *p* < 0.001). The MEDAS score was also significantly different between the two groups (7.4 ± 2.4 vs. 7.9 ± 2.5, *p* = 0.033).

**Table 2:** Sociodemographic characteristics, lifestyle habits and health status of the participants by the presence (depression) or absence (no depression) of self-reported depressive symptoms (*n* = 525).

Variables	Depression	No depression	*p*	Effect size
	(*n* = 127)	(*n* = 398)		
Categorical variables *n* (%)				
Sex			0.357	0.04
Female	78 (61.4)	226 (56.8)		
Male	49 (38.6)	172 (43.2)		
Regions			0.305	0.14
Beirut	7 (5.5)	47 (11.8)		
Beqaa	5 (3.9)	11 (2.8)		
Mount Lebanon	103 (81.1)	311 (78.1)		
North Lebanon	10 (7.9)	24 (6)		
South Lebanon	2 (1.6)	5 (1.3)		
Marital status			<0.001	0.33
Divorced/separated	0 (0)	6 (1.5)		
Married	8 (6.3)	109 (27.4)		
Single	117 (92.1)	282 (70.9)		
Widowed	2 (1.6)	1 (0.3)		
Current living status			<0.001	0.31
Live alone	7 (5.5)	28 (7)		
Live with another person	5 (3.9)	97 (24.4)		
Live with parents	115 (90.6)	273 (68.6)		
Education			0.008	0.19
College / undergraduate and above	101 (79.5)	353 (88.7)		
Elementary school and below	3 (2.4)	12 (3)		
High School	234 (18.1)	33 (8.3)		
Current occupation			<0.001	0.67
Unemployed	2 (1.6)	32 (8.0)		
Employed for full-time job	8 (6.3)	225 (56.5)		
Employed for part-time job	15 (11.8)	46 (11.6)		
Retired	0 (0)	6 (1.5)		
Studying	102 (80.3)	89 (22.4)		
Monthly income			0.001	0.22
$500–$1,000	94 (74)	226 (56.8)		
Less than $500	16 (12.6)	62 (15.6)		
More than $1,000	17 (13.4)	110 (27.6)		
E-cigarette smoking status			0.96	0.02
No	18 (14.2)	53 (13.3)		
Yes	75 (59.1)	240 (60.3)		
Never heard about EC	34 (26.8)	105 (26.4)		
BMI category			0.021	0.19
Healthy	67 (52.8)	232 (58.3)		
Obesity	12 (9.4)	49 (12.3)		
Overweight	35 (27.6)	103 (25.9)		
Underweight	13 (10.2)	14 (3.5)		
Continuous variables (median ± SD)				
Age	22 ± 5.8	25 ± 10.3	<0.001	0.264
Body mass index	23.2 ± 4.9	24.0 ± 4.5	0.19	0.134
MEDAS score 1	7.0 ± 2.4	8.0 ± 2.5	0.033	0.218
PHQ9 score 2	13.0 ± 4	5 ± 2.6	<0.001	3.038

### 3.3 Association of the levels of depression among participants with adherence to the Mediterranean diet

The distribution of the levels of the AMD by the presence (depression) or absence (No depression) of depressive symptoms is presented in Table 3. The diagram shows that about one-quarter (24%) of participants self-reported the presence of depressive symptoms with a PHQ-9 score greater than or equal to 10, as also shown in Table 1. The mean score of the PHQ-9 was higher in females than in males but was not statistically significant (7.51 ± 4.77 vs 6.86 ± 5.15, respectively; *p* = 0.137), as shown in Supplementary Table S2.

**Table 3:** Association between the presence (depression) or absence (no depression) of self-reported depressive symptoms and adherence to the Mediterranean diet (*n* = 525).

Variables	Depression	No depression	*p*	Effect size
	(*n* = 127)	(*n* = 398)		
MEDAS score	7.4 ± 2.4	7.9 ± 2.5	0.033	0.218
Levels of the AMD			0.143	0.12
High	25 (19.7)	109 (27.4)		
Low	14 (11)	30 (7.5)		
Medium	88 (69.3)	259 (65.1)		
Answers to the MEDAS items				
Olive oil main fat source			0.726	0.02
No	52 (40.9)	156 (39.2)		
Yes	75 (59.1)	242 (60.8)		
Olive oil ≥4 tbsp / d			0.695	0.02
No	68 (53.5)	221 (55.5)		
Yes	59 (46.5)	177 (44.5)		
Vegetables ≥2 servings / d			**< 0.001**	0.17
No	56 (44.1)	104 (26.1)		
Yes	71 (55.9)	294 (73.9)		
Fruits ≥3 servings/d			**0.007**	0.12
No	79 (62.2)	193 (48.5)		
Yes	48 (37.8)	205 (51.5)		
Red meat ≤1 serving/d			**0.049**	0.09
No	59 (46.5)	146 (36.7)		
Yes	68 (53.5)	252 (63.3)		
Butter/margarine ≤1 serving / d			0.551	0.03
No	67 (52.8)	222 (55.8)		
Yes	60 (40.7)	176 (44.2)		
Sweet beverages ≤1 serving / d			0.368	0.04
No	66 (52)	225 (56.50)		
Yes	61 (48)	173 (43.5)		
Red wine ≥7 glasses / w			0.811	0.01
No	98 (77.2)	303 (76.1)		
Yes	29 (22.8)	95 (23.9)		
Legumes ≥3 servings / w			0.115	0.07
No	35 (27.6)	83 (20.9)		
Yes	92 (72.4)	315 (79.1)		
Fish/seafood ≥3 servings / w			0.068	0.08
No	88 (69.3)	240 (60.3)		
Yes	39 (30.7)	158 (39.7)		
Sweets / pastries ≤3 servings / w			0.206	0.06
No	55 (43.3)	198 (49.7)		
Yes	72 (56.7)	200 (50.3)		
Nuts ≥3 servings/w			0.282	0.05
No	54 (42.5)	148 (37.2)		
Yes	73 (57.5)	250 (62.8)		
White meat preferences over red meat			0.953	0
No	38 (29.9)	118 (29.6)		
Yes	89 (70.1)	280 (70.4)		
Sofrito ≥2 eaten / w			0.188	0.06
No	24 (18.9)	56 (14.1)		
Yes	103 (81.1)	342 (85.9)		

Bold value indicate significant *p* value.

To examine the type of relationship between depression and adherence to the Mediterranean diet among our participants, we performed a Spearman correlation analysis. The results indicated a low, negative but significant relationship between the PHQ-9 score and the MEDAS score (*r* = −0.13, *p* = 0.003) and with age but not with BMI (Supplementary Figure S1).

### 3.4 Adherence to the Mediterranean diet characteristics of the participants by depression status

**Figure 1:**
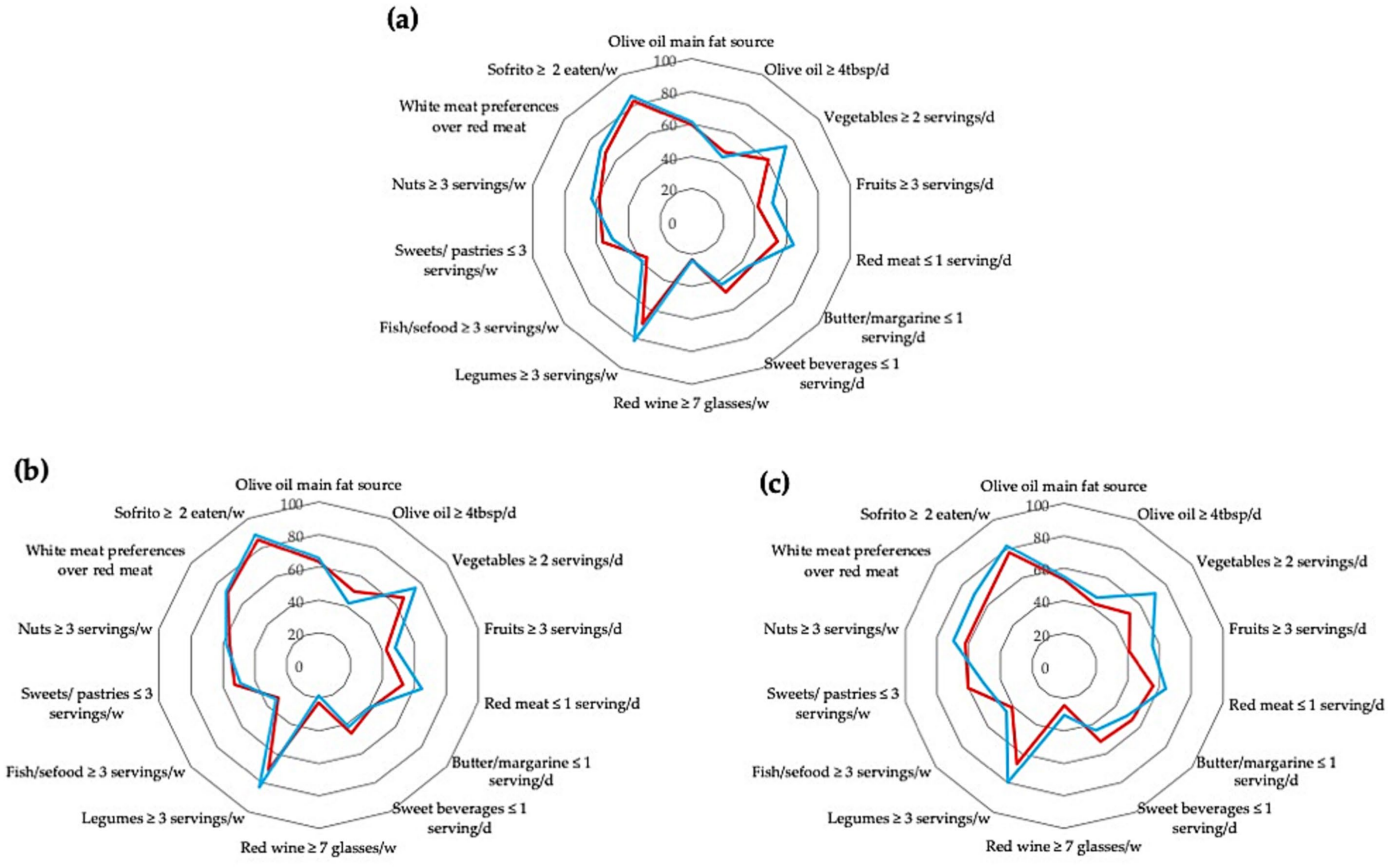
Compliance with the 14 items of the MEDAS questionnaire based on the presence (red line) and absence (blue line) of depressive symptoms in the total sample group **(a)**; in females **(b)** and in males **(c)**. The radar charts plot the frequency (0% in the center of the charts to 100% on the outermost ring) of each item of the MEDAS questionnaire along a separate axis. Tbsp, tablespoon; d, day; w, week.

Figure 1 indicated that medium AMD was the most prevalent dietary pattern in both groups, but Table 3 showed that it was not statistically significant [*χ*^2^(2) = 3.89, *p* = 0.143, Cramér’s V = 0.12]. However, we found a statistically significant relationship between vegetables ≥ 2 servings/d and depression with a medium-sized effect [*χ*^2^(1) = 14.66, *p* = <0.001, Cramér’s V = 0.17]. We also observed that “Fruits ≥3 servings /d” and “Red meat ≤1 serving /d” were significantly associated with self-reported depressive symptoms. These results were similar when stratified by sex, as shown in Figure 1.

We then performed a binary logistic regression analysis to explore the influence of the 14 food items from the MEDAS on depression. The unadjusted model presented in Table 4 indicated that of the 14 items of the MEDAS questionnaire, which served as independent variables, only vegetables ≥2 servings/d significantly predicted the presence of depressive symptoms among our participants. The beta coefficient (*β*) for the answer “Yes” for vegetables ≥2 servings/d negative (−0.64) suggests that the outcome of depression decreases. In other words, an intake of vegetables more than twice a day may decrease the odds of presenting depressive symptoms by 47.31% (*p* = 0.007). Although the coef. B for olive oil as the main fat source, fruits ≥3 servings/d, red meat ≤1 serving/d, legumes ≥3 servings/w, fish/seafood ≥3 servings/w, and sofrito ≥2 eaten/w were all negative, suggesting their preventive effect on the outcome depression, our results showed that they were not statistically significant. After adjustments for covariates, the trend of the association remained the same for the intake of vegetables ≥2 servings a day and reached borderline significance for an intake of red meat less than once a day in the fully adjusted model.

**Table 4:** Binary logistic regression analysis exploring the association between the presence of depressive symptoms (PHQ-9 ≥ 10) and each of the 14 items of the Mediterranean diet among the participants (*n* = 525).

Characteristics^*^	Model 1				Model 2				Model 3			
	*β*	*p*	COR	95% CI	*β*	*p*	AOR^1^	95% CI	*β*	*p*	AOR^2^	95% CI
Olive oil main fat source	−0.01	0.964	0.99	0.65–1.51	−0.06	0.799	0.94	0.6–1.48	0.08	0.768	1.08	0.64–1.82
Olive oil ≥4 tbsp / d	0.21	0.34	1.23	0.8–1.9	0.23	0.325	1.26	0.8–1.98	0.27	0.312	1.31	0.78–2.2
**Vegetables ≥2 servings / d**	**−0.64**	**0.007**	**0.53**	**0.33–0.84**	**−0.76**	**0.003**	**0.47**	**0.28–0.77**	**−0.66**	**0.021**	**0.52**	**0.29–0.91**
Fruits ≥3 servings / d	−0.27	0.252	0.76	0.48–1.21	−0.33	0.184	0.72	0.44–1.17	−0.4	0.157	0.67	0.38–1.17
Red meat ≤1 serving / d	−0.39	0.07	0.68	0.44–1.03	**−0.46**	**0.044**	**0.63**	**0.4–0.99**	−0.49	0.058	0.61	0.37–1.02
Butter/margarine ≤1 serving / d	0.12	0.611	1.12	0.72–1.76	0.13	0.597	1.13	0.71–1.81	0.27	0.323	1.31	0.77–2.22
Sweet beverages ≥1 serving / d	0.04	0.86	1.04	0.67–1.63	0.12	0.618	1.12	0.71–1.79	0.19	0.475	1.21	0.72–2.04
Red wine ≥7 glasses / w	0.15	0.56	1.17	0.69–1.96	0.2	0.478	1.22	0.7–2.12	0.34	0.298	1.4	0.74–2.64
Legumes ≥3 servings / w	−0.17	0.496	0.84	0.52–1.38	0.05	0.844	1.05	0.63–1.77	0.38	0.219	1.46	0.8–2.65
Fish/seafood ≥3 servings / w	−0.35	0.156	0.71	0.44–1.14	−0.39	0.135	0.68	0.41–1.13	−0.59	0.051	0.56	0.31–1
Sweets/pastries ≤3 servings / w	0.31	0.155	1.37	0.89–2.11	0.29	0.207	1.34	0.85–2.11	0.1	0.711	1.1	0.66–1.84
Nuts ≥3 servings / w	0.01	0.979	1.01	0.64–1.58	0.24	0.327	1.27	0.79–2.03	0.24	0.383	1.27	0.74–2.16
White meat preferences over red meat	0.07	0.767	1.07	0.67–1.71	0.08	0.734	1.09	0.67–1.77	0.14	0.621	1.15	0.66–1.99
Sofrito ≥2 eaten / w	−0.22	0.452	0.8	0.45–1.42	−0.34	0.276	0.71	0.39–1.31	−0.63	0.087	0.53	0.26–1.1

*Answer “No” to each item of the MEDAS questionnaire was used as reference category. Model 1: unadjusted with Crude Odds Ratio (COR). Model 2: Model 1 further adjusted for age, education, monthly income. Model 3: Model 2 further adjusted of current living conditions, current occupation, marital status and BMI. SE, Standard error; OR, Odds ratio; CI, Confidence interval; tbsp, tablespoon d, day; w, week. Bold value indicate significant *p* value.

## 4 Discussion

The present study aimed to determine the relationship between AMD and depressive symptoms as well as to identify specific food components of the MD and social factors that could serve as preventive and modifiable factors against depression in Lebanese adults. While the MD is a traditional dietary pattern that is integral to Lebanese cultural heritage and social practices, our findings showed that most participants moderately adhere to this diet. Our results confirmed an inverse correlation between AMD and depressive symptoms. To our knowledge, we were the first to identify that consuming more than two servings of vegetables per day significantly reduces the odds of depression among individuals in Lebanon. We also found that social factors like education, income, and marital and employment status may be predictors for depression.

### 4.1 Associations between AMD and mental health

Several studies have explored the association of mental health with dietary patterns in Lebanon. Our study findings concurred with the work of Ghernati et al. (44) who have shown that a poor diet, characterized by a higher intake of ultra-processed food, was significantly linked to higher depressive symptoms. In contrast, others did find only a relationship between AMD and anxiety, but not with depression (31, 33). This discrepancy may be explained by the different instrument used, the PHQ-4, and the nature and size of the study sample, as this cross-sectional study included only 200 university students (31).

Our results are in line with those reported in previous cross-sectional studies that have observed a decline in the AMD with an alarming shift from high to medium AMD (45), especially among the younger people (31, 33, 36, 37) while older people were following better dietary habits (46, 47). Interestingly, we have observed that age was inversely related to depressive symptoms. These data differ somehow from those reported in previous studies stating that depression is common in older people (48, 49); with higher prevalence among women as compared to men.

We have demonstrated that after adjustment with the sociodemographic covariates, the MEDAS item “vegetables ≥2 servings/d” was still significant in our regression model. This observation confirms the findings from other studies that have suggested the beneficial contribution of vegetable consumption to mental health in adults (50) or older people (51, 52). How a protective effect for mental health can be attributed to vegetables has not been fully elucidated, and data remain inconclusive. However, a recent review (53) identified vitamin C, a natural antioxidant essential for body homeostasis, as the most significant factor influencing the occurrence of depression in the general population. Indeed, Wang et al. (42) demonstrated a negative association between dietary vitamin C, particularly from vegetables, and the risk of depressive symptoms, especially among adult females and older people. Japanese investigators showed that dietary fiber intake from fruits and vegetables was associated with a lower likelihood of depressive symptoms (54). An Australian longitudinal study has shown that a daily higher intake of fruit ≥4 servings and vegetables ≥5 servings was systematically associated with lower odds of depression (55). Altogether, it is suggested that vitamin C and dietary fiber would exert their antidepressant effects through their antioxidant and anti-inflammatory properties (56, 57) and *via* the gut-brain axis (58, 59), respectively. We can hypothesize from our study that vitamin C may be one of the contributors in lowering the odds of depressive symptoms. The higher intake of some vegetables and fruits observed in our Lebanese cohort was similarly shown in another concurrently conducted study by our group in Lebanon, using a semi-food frequency questionnaire (35). Such a diet potentially rich in vitamin C may explain its beneficial effect against depression.

These findings have implications for the field of nutritional psychiatry and preventive mental health strategies, especially in social contexts like Lebanon. It suggests that dietary interventions could serve as accessible and cost-effective adjuncts to traditional psychiatric treatments. Mental health practitioners should consider incorporating nutritional assessments and dietary counselling into routine clinical practice (60), especially for patients presenting with mild to moderate depressive symptoms or those at risk for depression. Given Lebanon’s ongoing socioeconomic challenges and the cultural significance of the MD in Lebanese society, promoting adherence to traditional dietary patterns can represent a culturally appropriate preventive strategy (14). Furthermore, the identification of specific dietary components, particularly vegetable consumption, provides clinicians with concrete, actionable recommendations that can be easily integrated into dietary interventions.

### 4.2 Correlation between social factors and mental health

In terms of the relation between social factors and depression, the study found several variables associated with depression, namely economic variables, education level, and marital status. First, regarding the economic factors and education level, our study echoes the results of previous ones that underline their protective role for mental health. This relationship is especially true in circumstances of economic struggles, similar to those of Lebanon (6). Indeed, research has often supported that people with lower socioeconomic status are more vulnerable to mental health concerns (61). Second, marital status was associated with depression. This can be explained by the protective role of spouse support, as previous studies have indicated that support from one’s partner is beneficial for mental health (62).

The higher depression levels observed among females in our study sample are in accordance with previous studies, which did not systematically imply diet as a contributing factor for depression. Instead, they emphasized the role of other factors, such as interpersonal relationships, fatigue, goal-orientated factors, pre/postmenstrual, postpartum and postmenopausal conditions, to explain the depressive symptoms (63–65). Additionally, gender norms and sociocultural factors may also interact with biological variables, thus increasing the risk of depression among women (66). Indeed, women are more likely to suffer from depression, despite their better dietary habits resulting from healthier food choices (37, 47, 67). These data highlight the importance of sociodemographic factors in explaining this observation. Nonetheless, in women with depression, depressive symptoms decreased as their dietary compliance improved (47, 67). Further investigation in this matter is underway in our unit.

### 4.3 Limitations

First, this study was observational; therefore, we could not determine whether variations in AMD precede the occurrence of depressive symptoms and vice versa. Future research employing a longitudinal design would be necessary to further clarify these relationships. This limitation illustrates the complexity of the study of dietary influences on mental health. Second, our study utilized the snowball sampling method, which is known to exhibit self-selection bias and non-response bias. While participants may have recruited others from their own social networks, some potential participants may have ignored the invitation or belonged to hard-to reach groups. Third, given that this study relied on self-report instruments, the BMI was merely an estimate of the actual body weight status of our participants. Additionally, it was not possible to quantify the specific types of vegetables or their respective macronutrient and micronutrient compositions. This limitation highlights the need for more detailed dietary assessments that not only consider food groups but also analyze the nutritional content of individual foods. Finally, as discussed previously, the variability among regions for AMD impedes the transferability of our data to another region or country. Future research should further explore these regional differences to enhance the applicability of findings across diverse contexts.

## 5 Conclusion

Overall, our data indicate that AMD and daily intake of more than two servings of vegetables can help reduce depressive symptoms in Lebanese adults. These findings underscore the potential of nutritional interventions as a complementary approach to traditional mental health care for depression. By integrating dietary recommendations with attention to social factors that present a risk for depression, clinicians can adopt a more holistic approach to mental health treatment and prevention.

## Data Availability

The raw data supporting the conclusions of this article will be made available by the corresponding author, upon reasonable request.

## References

[1] Sun X Yon DK Nguyen TT Tanisawa K Son K Zhang L . Dietary and other lifestyle factors and their influence on non-communicable diseases in the Western Pacific region. Lancet Reg Health West Pac. (2024) 43:100842. doi: 10.1016/j.lanwpc.2023.100842, 38456094 PMC10920053

[2] Tafuri D Latino F. Association of Dietary Intake with chronic disease and human health. Nutrients. (2025) 17:446. doi: 10.3390/nu17030446, 39940304 PMC11821025

[3] Zhang Y Jia X Yang Y Sun N Shi S Wang W. Change in the global burden of depression from 1990-2019 and its prediction for 2030. J Psychiatr Res. (2024) 178:16–22. doi: 10.1016/j.jpsychires.2024.07.054, 39106579

[4] Chan VKY Leung MYM Chan SSM Yang D Knapp M Luo H . Projecting the 10-year costs of care and mortality burden of depression until 2032: a Markov modelling study developed from real-world data. Lancet Reg Health West Pac. (2024) 45:101026. doi: 10.1016/j.lanwpc.2024.101026, 38352243 PMC10862399

[5] Al-Khalil ZM El Sheikh WG Lababidi GH Shehayeb EO Ghanime PM Talih FR . Impact of socioeconomic and political stressors on mental health: a cross-sectional study on university students in Lebanon. BMC Med Educ. (2025) 25:91. doi: 10.1186/s12909-025-06701-1, 39827125 PMC11743072

[6] El Murr P Rahme E Younes RS Assaf R Zalaket N. Mental health during the Lebanese economic crisis: association between financial well-being, anxiety, and depression. Int J Soc Psychiatry. (2025). doi: 10.1177/00207640251359059, 40819266 PMC12946224

[7] Karam EG El-Jamal M Osman R Toukan S Mouawad GI Al Barathie J. The aftermath of multiple trauma on a nation: unraveling Lebanon’s unique mental health struggle. Front Psych. (2025) 15:1444245. doi: 10.3389/fpsyt.2024.1444245, 39876996 PMC11773410

[8] Cui L Li S Wang S Wu X Liu Y Yu W . Major depressive disorder: hypothesis, mechanism, prevention and treatment. Signal Transduct Target Ther. (2024) 9:30. doi: 10.1038/s41392-024-01738-y, 38331979 PMC10853571

[9] Picone P Girgenti A Buttacavoli M Nuzzo D. Enriching the Mediterranean diet could nourish the brain more effectively. Front Nutr. (2024) 11:1489489. doi: 10.3389/fnut.2024.1489489, 39664911 PMC11631615

[10] Adan RAH Van Der Beek EM Buitelaar JK Cryan JF Hebebrand J Higgs S . Nutritional psychiatry: towards improving mental health by what you eat. Eur Neuropsychopharmacol. (2019) 29:1321–32. doi: 10.1016/j.euroneuro.2019.10.011, 31735529

[11] Peedicayil J. The role of epigenetics in social psychiatry. Int J Soc Psychiatry. (2017) 63:14–20. doi: 10.1177/0020764016677556, 27856950

[12] Guasch-Ferré M Willett WC. The Mediterranean diet and health: a comprehensive overview. J Intern Med. (2021) 290:549–66. doi: 10.1111/joim.13333, 34423871

[13] Yesildemir O Guldas M Boqué N Calderón-Pérez L Degli Innocenti P Scazzina F . Adherence to the Mediterranean diet among families from four countries in the Mediterranean basin. Nutrients. (2025) 17:1157. doi: 10.3390/nu17071157, 40218915 PMC11990228

[14] Trajkovska Petkoska A Ognenoska V Trajkovska-Broach A. Mediterranean diet: from ancient traditions to modern science—a sustainable way towards better health, wellness, longevity, and personalized nutrition. Sustainability. (2025) 17:4187. doi: 10.3390/su17094187

[15] Kiani AK Medori MC Bonetti G Aquilanti B Velluti V Matera G . Modern vision of the Mediterranean diet. J Prev Med Hyg. (2022) 63:E36–43. doi: 10.15167/2421-4248/JPMH2022.63.2S3.2745, 36479477 PMC9710405

[16] Morris L Bhatnagar D. The Mediterranean diet. Curr Opin Lipidol. (2016) 27:89–91. doi: 10.1097/MOL.0000000000000266, 26655296

[17] El Rayess Y Nehme N Azzi-Achkouty S Julien SG. Wine phenolic compounds: chemistry, functionality and health benefits. Antioxidants. (2024) 13:1312. doi: 10.3390/antiox13111312, 39594454 PMC11591289

[18] Willett W Skerrett PJ Giovannucci EL. Eat, drink, and be healthy: the Harvard Medical School guide to healthy eating. New York, NY: Free Press (2017).

[19] Maddaloni L Donini LM Gobbi L Muzzioli L Vinci G. Essential amino acids and fatty acids in novel foods: emerging nutritional sources and implications. Dietetics. (2025) 4:14. doi: 10.3390/dietetics4020014

[20] Boujelbane MA Ammar A Salem A Kerkeni M Trabelsi K Bouaziz B . Regional variations in Mediterranean diet adherence: a sociodemographic and lifestyle analysis across Mediterranean and non-Mediterranean regions within the MEDIET4ALL project. Front Public Health. (2025) 13:1596681. doi: 10.3389/fpubh.2025.1596681 40556924 PMC12185284

[21] Conti S Perdixi E Bernini S Jesuthasan N Severgnini M Prinelli F. Adherence to Mediterranean diet is inversely associated with depressive symptoms in older women: findings from the NutBrain study. Br J Nutr. (2024) 131:1892–901. doi: 10.1017/S0007114524000461, 38361447

[22] Currenti W Godos J Alanazi AM Lanza G Ferri R Caraci F . Dietary fats and depressive symptoms in Italian adults. Nutrients. (2023) 15:675. doi: 10.3390/nu15030675, 36771380 PMC9919703

[23] Argyropoulos K Machini E. Adherence to Mediterranean diet and risk of depression later in life. A cross sectional study in East Attica, Greece. Glob Psychiatry Arch. (2020) 2:201–10. doi: 10.52095/gpa.2020.1335

[24] Gregório MJ Rodrigues AM Eusébio M Sousa RD Dias S André B . Dietary patterns characterized by high meat consumption are associated with other unhealthy life styles and depression symptoms. Front Nutr. (2017) 4:25. doi: 10.3389/fnut.2017.00025, 28660194 PMC5469910

[25] Hernández-Galiot A Goñi I. Adherence to the Mediterranean diet pattern, cognitive status and depressive symptoms in an elderly non-institutionalized population. Nutr Hosp. (2017) 34:338. doi: 10.20960/nh.360 28421787

[26] Andrade V Jorge R García-Conesa M-T Philippou E Massaro M Chervenkov M . Mediterranean diet adherence and subjective well-being in a sample of Portuguese adults. Nutrients. (2020) 12:3837. doi: 10.3390/nu12123837, 33339084 PMC7765516

[27] Deligiannidou G-E Philippou E Vasiari E De Andrade VL Massaro M Chervenkov M . Exploring the relationship between Mediterranean diet adherence and subjective well-being among Greek and Cypriot adults. Nutrients. (2024) 16:1238. doi: 10.3390/nu16081238, 38674928 PMC11054782

[28] Jiménez-López E Mesas AE Visier-Alfonso ME Pascual-Morena C Martínez-Vizcaíno V Herrera-Gutiérrez E . Adherence to the Mediterranean diet and depressive, anxiety, and stress symptoms in Spanish adolescents: results from the EHDLA study. Eur Child Adolesc Psychiatry. (2024) 33:2637–46. doi: 10.1007/s00787-023-02351-0, 38170283

[29] Bayram HM Aydın AG Okur H Karalı AE Öztürkcan A. The relationship between dietary polyphenol intake and adherence to the Mediterranean diet, mental health, and sleep quality among Turkish adults: a cross-sectional study. Food and Health. (2024) 10:262–72. doi: 10.3153/FH24025

[30] Achour Y Lucas G Iceta S Boucekine M Rahmati M Berk M . Dietary patterns and major depression: results from 15,262 participants (international alimental study). Nutrients. (2025) 17:1583. doi: 10.3390/nu17091583, 40362892 PMC12073559

[31] El Mikkawi H El Khoury C Rizk R. Adherence to the Mediterranean diet and mental health among university students in Lebanon. Appl Food Res. (2024) 4:100435. doi: 10.1016/j.afres.2024.100435

[32] AL-Basri H Fallows S. The Mediterranean diet is not related to depression, anxiety, and stress among young university students in the UK: a cross-sectional study. Glob Psychiatry Arch. (2023) 6:37–50. doi: 10.52095/gpa.2023.5941.1065

[33] Allcock L Mantzioris E Villani A. Adherence to a Mediterranean diet is inversely associated with anxiety and stress but not depression: a cross-sectional analysis of community-dwelling older Australians. Nutrients. (2024) 16:366. doi: 10.3390/nu16030366, 38337651 PMC10857277

[34] Farran N. Mental health in Lebanon: tomorrow’s silent epidemic. Ment Health Prev. (2021) 24:200218. doi: 10.1016/j.mhp.2021.200218, 34660191 PMC8503814

[35] Bou Chacra CJ Julien SG. Use of a food frequency questionnaire for the estimation of gut microbiota composition based on dietary patterns and its association with irritable bowel syndrome symptoms in the Lebanese adult population: a cross-sectional study. Adv Public Health. (2024) 2024:1–14. doi: 10.1155/2024/6962855

[36] El Hajj JS Julien SG. Factors associated with adherence to the Mediterranean diet and dietary habits among university students in Lebanon. J Nutr Metab. (2021) 2021:6688462. doi: 10.1155/2021/6688462, 33564473 PMC7850855

[37] El Khoury CN Julien SG. Inverse association between the Mediterranean diet and COVID-19 risk in Lebanon: a case-control study. Front Nutr. (2021) 8:707359. doi: 10.3389/fnut.2021.707359, 34395500 PMC8363114

[38] Martínez-González MA García-Arellano A Toledo E Salas-Salvadó J Buil-Cosiales P Corella D . A 14-item mediterranean diet assessment tool and obesity indexes among high-risk subjects: the PREDIMED trial. PLoS One. (2012) 7:e43134. doi: 10.1371/journal.pone.0043134, 22905215 PMC3419206

[39] Schröder H Fitó M Estruch R Martínez-González MA Corella D Salas-Salvadó J . A short screener is valid for assessing Mediterranean diet adherence among older Spanish men and women. J Nutr. (2011) 141:1140–5. doi: 10.3945/jn.110.135566, 21508208

[40] Kroenke K Spitzer RL Williams JBW. The PHQ-9: validity of a brief depression severity measure. J Gen Intern Med. (2001) 16:606–13. doi: 10.1046/j.1525-1497.2001.016009606.x, 11556941 PMC1495268

[41] Glavin D Maekawa E Grua EM Nakamura CA Scazufca M Araya R . Other PHQ-9 item pairings are better than the PHQ-2: a machine learning analysis. Procedia Comput Sci. (2022) 206:101–10. doi: 10.1016/j.procs.2022.09.089

[42] Wang A Luo J Zhang T Zhang D. Dietary vitamin C and vitamin C derived from vegetables are inversely associated with the risk of depressive symptoms among the general population. Antioxidants. (2021) 10:1984. doi: 10.3390/antiox10121984, 34943087 PMC8750333

[43] Summaka M Zein H Abbas LA Elias C Elias E Fares Y . Validity and reliability of the Arabic patient health Questionnaire-9 in patients with spinal cord injury in Lebanon. World Neurosurg. (2019) 125:e1016–22. doi: 10.1016/j.wneu.2019.01.234, 30771543

[44] Ghernati L Tamim H Chokor FAZ Taktouk M Assi B Nasreddine L . Processed and ultra-processed foods are associated with depression and anxiety symptoms in a cross-sectional sample of urban Lebanese adults. Nutr Res. (2025) 133:172–89. doi: 10.1016/j.nutres.2024.11.011, 39764859

[45] Cardamone E Iacoponi F Di Benedetto R Lorenzoni G Di Nucci A Zobec F . Adherence to Mediterranean diet and its main determinants in a sample of Italian adults: results from the ARIANNA cross-sectional survey. Front Nutr. (2024) 11:1346455. doi: 10.3389/fnut.2024.1346455, 38476598 PMC10927747

[46] Marques-Vidal P Waeber G Vollenweider P Guessous I. Socio-demographic and lifestyle determinants of dietary patterns in French-speaking Switzerland, 2009–2012. BMC Public Health. (2018) 18:131. doi: 10.1186/s12889-018-5045-1, 29329572 PMC5766995

[47] Nowicki GJ Polak M Ślusarska B Czernecki K. The relationship between diet and the occurrence of depressive symptoms in a community example with high rates of social deprivation: a cross-sectional study. Nutrients. (2023) 15:3778. doi: 10.3390/nu15173778, 37686809 PMC10489963

[48] Alfaifi F. Elmahdy M. El-Setouhy M. A. Alfaifi A. 2024 Prevalence of depression among older adults visiting the primary healthcare centers in Jizan city, Saudi Arabia: an analytical cross-sectional study Cureus doi: 10.7759/cureus.52847 16 e52847 38406060 PMC10884985

[49] Peng X Zhang S You L Hu W Jin S Wang J. Prevalence and correlates of depression and anxiety symptoms among older adults in Shenzhen, China: a cross-sectional population-based study. BMJ Open. (2024) 14:e077078. doi: 10.1136/bmjopen-2023-077078, 38341212 PMC10862290

[50] Tuck N-J Farrow C Thomas JM. Assessing the effects of vegetable consumption on the psychological health of healthy adults: a systematic review of prospective research. Am J Clin Nutr. (2019) 110:196–211. doi: 10.1093/ajcn/nqz080, 31152539

[51] Głąbska D Guzek D Groele B Gutkowska K. Fruit and vegetable intake and mental health in adults: a systematic review. Nutrients. (2020) 12:115. doi: 10.3390/nu12010115, 31906271 PMC7019743

[52] Matison AP Flood VM Lam BCP Lipnicki DM Tucker KL Preux P-M . Associations between fruit and vegetable intakes and incident depression in middle-aged and older adults from 10 diverse international longitudinal cohorts. J Affect Disord. (2024) 359:373–81. doi: 10.1016/j.jad.2024.05.096 38788860

[53] Alberts A Moldoveanu E-T Niculescu A-G Grumezescu AM. Vitamin C: a comprehensive review of its role in health, disease prevention, and therapeutic potential. Molecules. (2025) 30:748. doi: 10.3390/molecules30030748, 39942850 PMC11820684

[54] Miki T Eguchi M Kurotani K Kochi T Kuwahara K Ito R . Dietary fiber intake and depressive symptoms in Japanese employees: the Furukawa nutrition and health study. Nutrition. (2016) 32:584–9. doi: 10.1016/j.nut.2015.11.014, 26810963

[55] Dharmayani PNA Mishra GD Mihrshahi S. Fruit and vegetable consumption and depression symptoms in young women: results from 1973 to 1978 cohort of the Australian longitudinal study on women’s health. Eur J Nutr. (2022) 61:4167–78. doi: 10.1007/s00394-022-02926-8, 35864339 PMC9596510

[56] Ferreira NR Vitorino C Fortuna A. From antioxidant to neuromodulator: the role of ascorbate in the management of major depression disorder. Biochem Pharmacol. (2022) 206:115300. doi: 10.1016/j.bcp.2022.115300, 36261067

[57] Gęgotek A Skrzydlewska E. Antioxidative and anti-inflammatory activity of ascorbic acid. Antioxidants. (2022) 11:1993. doi: 10.3390/antiox11101993, 36290716 PMC9598715

[58] Aslam H Lotfaliany M So D Berding K Berk M Rocks T . Fiber intake and fiber intervention in depression and anxiety: a systematic review and meta-analysis of observational studies and randomized controlled trials. Nutr Rev. (2024) 82:1678–95. doi: 10.1093/nutrit/nuad143, 38007616 PMC11551482

[59] Sim M Hong S Jung MH Choi EY Hwang G-S Shin D-M . Gut microbiota links vitamin C supplementation to enhanced mental vitality in healthy young adults with suboptimal vitamin C status: a randomized, double-blind, placebo-controlled trial. Brain Behav Immun. (2025) 128:179–91. doi: 10.1016/j.bbi.2025.03.032, 40187667

[60] Koshimoto S Kuboki N Gunji C Fujiwara M Hayashi H Moriya H . Nutritional counseling needs of patients with mental disorders in psychiatric care: a cross-sectional survey. Int J Soc Psychiatry. (2023) 69:1693–703. doi: 10.1177/00207640231174366, 37218288

[61] Azizabadi Z Aminisani N Emamian MH. Socioeconomic inequality in depression and anxiety and its determinants in Iranian older adults. BMC Psychiatry. (2022) 22:761. doi: 10.1186/s12888-022-04433-w, 36471352 PMC9721087

[62] Rostami A Ghazinour M Richter J. Marital satisfaction: the differential impact of social support dependent on situation and gender in medical staff in Iran. Global J Health Sci. (2013) 5:p151. doi: 10.5539/gjhs.v5n4p151, 23777731 PMC4776803

[63] Albert PR. Why is depression more prevalent in women? J Psychiatry Neurosci. (2015) 40:219–21. doi: 10.1503/jpn.150205, 26107348 PMC4478054

[64] Tang J Zhang T. Causes of the male-female ratio of depression based on the psychosocial factors. Front Psychol. (2022) 13:1052702. doi: 10.3389/fpsyg.2022.1052702, 36467188 PMC9709267

[65] Zhou W Hei B Liu Z Liu Y Ding Z Li M. Global temporal trends in depression incidence among women of childbearing age: a 30-year analysis and projections to 2030. Soc Sci Med. (2025) 372:118005. doi: 10.1016/j.socscimed.2025.118005, 40147333

[66] Koenig LR Blum RW Shervington D Green J Li M Tabana H . Unequal gender norms are related to symptoms of depression among young adolescents: a cross-sectional, cross-cultural study. J Adolesc Health. (2021) 69:S47–55. doi: 10.1016/j.jadohealth.2021.01.023, 34217459

[67] Nouri Saeidlou S Kiani A Ayremlou P. Association between dietary patterns and major depression in adult females: a case-control study. J Res Health Sci. (2021) 21:e00506. doi: 10.34172/jrhs.2021.37, 34024764 PMC8957692

