# Supplementary material for: Examining the association between the Mediterranean diet and depression: a cross-sectional study in Lebanon
Source: Front Nutr. 2025 Dec 17;12:1692981. doi: 10.3389/fnut.2025.1692981 (PMC12753428; doi:10.3389/fnut.2025.1692981)
Supplement: Supplementary file 1 [file Table_1.DOCX]

Supplementary Material

**Supplementary Table 1:** Description of variables with corresponding measurement scales and coding schemes.

| **Variables** | **Measurement Scale** | **Coding Scheme** |
| --- | --- | --- |
| **Continuous Variables** |  |  |
| Age | Continuous (Ratio) | Actual age in years |
| BMI (Body Mass Index) | Continuous (Ratio) | Calculated as $\frac{Weight (kg)}{Height^{2} (m^{2})}$ |
| MEDAS score | Continuous (Interval) | 0 to 14 scale |
| PHQ-9 score | Continuous (Interval) | 0 to 27 scale |
| **Categorical Variables** |  |  |
| Gender | Nominal | 1= Male/ 2= Female |
| Region | Nominal | 1= Beirut/ 2= Beqaa/ 3= Mount Lebanon/ 4= North Lebanon/ 5= South Lebanon |
| Marital status | Nominal | 1= Single/ 2= Married/ 3= Divorced/Separated/Widowed |
| Current living status | Nominal | 1= Live alone/ 2= Live with parents/ 3= Live with another person |
| Education | Ordinal | 1= Elementary school and below/ 2= High school/ 3= College/ Undergraduate and above |
| Occupation | Nominal | 1= Employed for full-time/ 2= Employed for part-time/ 3= retired/ 4= studying |
| Monthly income | Ordinal | 1= Less than 500$/ 2= 500$-1000$/ 3= More than 1000$ |
| E-cigarette smoking status | Nominal | 0= No/ 1=Yes/ 2= Never heard about E-cigarettes |
| BMI category | Ordinal | 1= Healthy/ 2=Overweight/ 3=Obese/ 4=Underweight |
| AMD (MEDAS score) | Ordinal | 1= Low/ 2= Moderate/ 3= High |
| PHQ-9 (Depression score) | Ordinal | 1= Minimal depression/ 2= Mild depression/ 3= Moderate depression/ 4= Moderately severe depression/ 5= Severe depression |

**Supplementary Table 2:** Sociodemographic characteristics, lifestyle habits, and health status of the participants by sex.

| Variables | Male | Female | *p* | Effect size^c^ |
| --- | --- | --- | --- | --- |
|  | (n = 304) | (n = 221) |  |  |
| Continuous (median ± SD) |  |  |  |  |
| Age | 24.0 ± 9.9 | 24 ± 9.6 | < 0.001 | 0.614 |
| BMI | 25.5 ± 4.4 | 22.4 ± 4.4 | 0.19 | 0.134 |
| MEDAS score | 8.0 ± 2.4 | 8.0 ± 2.4 | 0.033 | 0.218 |
| PHQ-9 score | 6 ± 5.1 | 7 ± 4.8 | < 0.001 | 3.038 |
| Categorical^1^ n (%) |  |  |  |  |
| Gender |  |  | *0.357* | 0.04 |
| Female | 78 (61.4) | 226 (56.8) |  |  |
| Male | 49 (38.6) | 172 (43.2) |  |  |
| Regions |  |  | 0.305 | 0.14 |
| Beirut | 7 (5.5) | 47 (11.8) |  |  |
| Beqaa | 5 (3.9) | 11 (2.8) |  |  |
| Mount Lebanon | 103 (81.1) | 311 (78.1) |  |  |
| North Lebanon | 10 (7.9) | 24 (6) |  |  |
| South Lebanon | 2 (1.6) | 5 (1.3) |  |  |
| Marital status |  |  | < 0.001 | 0.33 |
| Divorced/Separated | 0 (0) | 6 (1.5) |  |  |
| Married | 8 (6.3) | 109 (27.4) |  |  |
| Single | 117 (92.1) | 282 (70.9) |  |  |
| Widowed | 2 (1.6) | 1 (0.3) |  |  |
| Current living status |  |  | < 0.001 | 0.31 |
| Live alone | 7 (5.5) | 28 (7) |  |  |
| Live with another person | 5 (3.9) | 97 (24.4) |  |  |
| Live with parents | 115 (90.6) | 273 (68.6) |  |  |
| Education |  |  | 0.008 | 0.19 |
| College / undergraduate and above | 101 (79.5) | 353 (88.7) |  |  |
| Elementary school and below | 3 (2.4) | 12 (3) |  |  |
| High school | 23 (18.1) | 33 (8.3) |  |  |
| Monthly income |  |  | 0.001 | 0.22 |
| $500 - $1000 | 94 (74) | 226 (56.8) |  |  |
| Less than $500 | 16 (12.6) | 62 (15.6) |  |  |
| More than $1000 | 17 (13.4) | 110 (27.6) |  |  |
| E-cigarette smoking status |  |  | 0.96 | 0.02 |
| No | 18 (14.2) | 53 (13.3) |  |  |
| Yes | 75 (59.1) | 240 (60.3) |  |  |
| Never heard about EC | 34 (26.8) | 105 (26.4) |  |  |
| BMI category |  |  | 0.021 | 0.19 |
| Healthy | 67 (52.8) | 232 (58.3) |  |  |
| Obesity | 12 (9.4) | 49 (12.3) |  |  |
| Overweight | 35 (27.6) | 103 (25.9) |  |  |
| Underweight | 13 (10.2) | 14 (3.5) |  |  |
| AMD (MEDAS score) |  |  | 0.143 | 0.12 |
| High | (27.4) | 109 (27.4) |  |  |
| Low | (7.5) | 30 (7.5) |  |  |
| Medium | (65.1) | 259 (65.1) |  |  |
| *a student's t-test.* | | | |  |
| *b. A chi-squared test was performed when all expected cell frequencies were greater than 5.* | | | | |
| *c Effect size represented by Cohen's d value* | | | | |
| *d Cramer's V was used to calculate the effect size.* | | | | |

**Supplementary Figure 1.** Linear regression analysis between PHQ9 score and age (a) and BMI (b)
